# Outcomes of Total Hip Arthroplasty in Patients with Osteonecrosis of the Femoral Head Following Surgical Treatment of Brain Tumors

**DOI:** 10.3390/jcm8101703

**Published:** 2019-10-16

**Authors:** Seung-Jae Lim, Chan-Woo Park, Dong-Uk Kim, Kwangjoon Han, Minkyu Seo, Young-Wan Moon, Jung-Il Lee, Youn-Soo Park

**Affiliations:** 1 Department of Orthopedic Surgery, Samsung Medical Center, Sungkyunkwan University School of Medicine, Seoul 06351, Korea; limsj70@gmail.com (S.-J.L.); existcwp@gmail.com (C.-W.P.); osdongukkim@gmail.com (D.-U.K.); hkj7603@gmail.com (K.H.); samil4406@gmail.com (M.S.); ywmoon@skku.edu (Y.-W.M.); 2 Department of Neurosurgery, Samsung Medical Center, Sungkyunkwan University School of Medicine, Seoul 06351, Korea; jilee@skku.edu

**Keywords:** total hip arthroplasty, osteonecrosis of the femoral head, corticosteroid, brain tumor, outcome, complication

## Abstract

Corticosteroids have been widely used in patients with brain tumors to reduce tumor-associated edema and neurological deficits. This study examined the outcomes of total hip arthroplasty (THA) in patients with osteonecrosis of the femoral head (ONFH) following brain tumor surgery. We identified 34 THAs performed in 26 patients with steroid-induced ONFH among 9254 patients undergoing surgical treatment for primary brain tumors. After propensity score matching with demographics, 68 THAs (52 patients) in ONFH unrelated to brain tumors were selected as the control group. At the time of THA, 54% of brain tumor patients had neurological sequelae and 46% had adrenal insufficiency. After THA, patients with brain tumor required longer hospital stay, reported a lower functional score, and showed a higher rate of heterotopic ossification compared to the control group. However, hip pain score improved significantly after THA in the brain tumor group, and did not differ from that of the control group (*P*-value = 0.168). Major complication rates were similar (2.9% and 1.5% for the brain tumor and control groups, respectively; *P*-value = 1.000), and implant survivorships were not different at 7 years (100% and 98.1% for the brain tumor and control groups, respectively; *P*-value = 0.455). Our findings suggest that THA can be safely performed to reduce hip pain in patients with steroid-induced ONFH after surgical treatment of primary brain tumors.

## 1. Introduction

Corticosteroids have been commonly used in patients with brain tumors to control peritumoral edema and neurological symptoms [1,2,3]. Dexamethasone is the drug of choice in modern neuro-oncology due to its potent glucocorticoid activity and weak mineralocorticoid effects [4]. The optimal dose of dexamethasone in the perioperative period of neurosurgery is 10–32 mg/day, which is considerably higher than the usual therapeutic dose [5]. Although short-term use is generally recommended, it requires a substantial tapering period to avoid secondary adrenal insufficiency. Moreover, surgical resection of sellar lesions (e.g., pituitary adenoma and craniopharyngioma) frequently results in dysfunction of the hypothalamic–pituitary–adrenal (HPA) axis, which necessitates long-term steroid replacement [6,7,8].

Prolonged treatment with high doses of corticosteroids increases the frequency of systemic adverse events. A serious corticosteroid-induced complication in the musculoskeletal system is osteonecrosis of the femoral head (ONFH). A large necrotic lesion frequently causes severe hip pain, which significantly impairs the quality of life. Although total hip arthroplasty (THA) has become the most successful surgical option for treating painful ONFH, a proportion of early failure in THA is inevitable [9,10]. The three leading causes of reoperation in contemporary THA are recurrent dislocation, periprosthetic joint infection (PJI), and periprosthetic femoral fracture (PFF), all of which are still challenging situations.

Patients with primary brain tumor have several features that make orthopaedic surgeons reluctant to perform THA. There may be increased risk for PJI, as a large number of them are chronic steroid users [11,12,13]. There are also concerns regarding the high prevalence of neurological sequelae (e.g., seizures and cognitive impairment) after brain tumor surgery. Poor compliance with postoperative range-of-motion (ROM) restriction can increase the incidence of dislocation [14,15], and frequent falls during rehabilitation can eventually lead to PFF [16]. Therefore, we hypothesized that patients with resected brain tumors would have a higher rate of complications and report a lower functional score after THA, compared to patients without brain tumors.

The purpose of this study was to investigate patient characteristics, complication rates, clinical scores, and radiographic results of THA performed in ONFH following surgical resection of primary brain tumors. We also compared the results with those in a matched control group of ONFH unrelated to brain tumors.

## 2. Material and Methods

### 2.1. Patient Cohort

This study was performed with the approval of our institutional review board. A total of 9254 patients underwent surgical removal of primary brain tumors at a tertiary referral center from January 2003 to December 2014. By searching with the medical procedure code, we identified 33 patients (42 hips) who received hip arthroplasty after brain tumor surgery at the same center. Those who had corticosteroid therapy during the perioperative period of brain tumor surgery and subsequently underwent THA due to ONFH were included. Patients with diagnosis other than ONFH, those with diagnosis of ONFH prior to brain tumor surgery, and those with resurfacing arthroplasty were excluded from the study. After exclusion of these cases, 29 patients (37 hips) remained. Of these, two patients (two hips) died due to tumor progression, while one patient (one hip) was lost to follow-up before reaching a minimum of 2 years. There were no cases of revision surgery or complications in these patients by the last follow-up.

**Figure 1:**
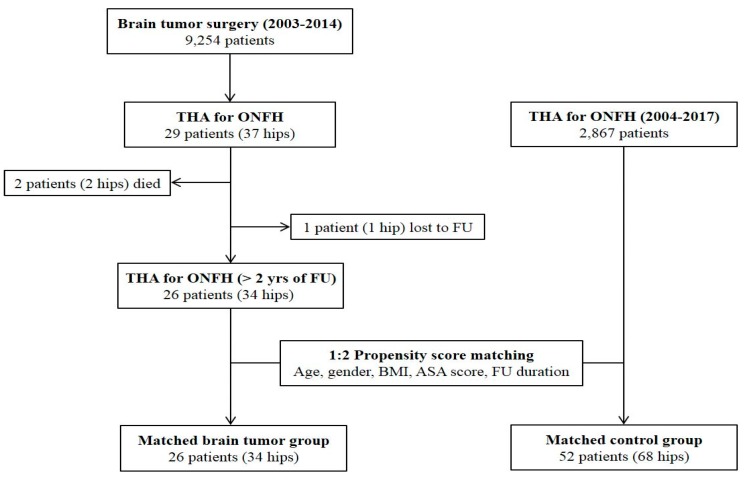
Patient flow diagram. THA, total hip arthroplasty; ONFH, osteonecrosis of the femoral head; FU, follow-up; BMI, body mass index; ASA, American Society of Anesthesiologists.

The remaining 26 patients (34 hips) with brain tumors were included in the study population. Among them, 8 patients underwent simultaneous bilateral THA or sequential bilateral THA due to ONFH involved in both hips. All THAs were performed between April 2004 and February 2017. During the same period, a total of 2867 patients underwent primary THA due to ONFH. We set the matched control group among these patients using the 1:2 matching technique. After generation of the propensity score with age, sex, body mass index (BMI), American Society of Anesthesiologists (ASA) score, and follow-up duration, 52 patients (68 hips) were selected as the control group (Figure 1). All surgical outcomes were compared between the two matched groups.

### 2.2. Baseline Evaluation

Brain tumors were classified according to their anatomical locations and histopathological results. The presence of neurological symptoms was identified by reviewing medical records at the time of THA. We defined cognitive impairment as a Mini-Mental State Examination (MMSE) score of ≤24 points [17]. We considered the presence of seizures if there was any focal or generalized epileptic episode within 3 months prior to THA. Hemiparesis was defined as partial paralysis or muscle weakness on one side of the body. Visual disturbance included a decrease in vision, a visual field defect, and oculomotor dysfunction. We examined each dose and duration of corticosteroid treatment from the time of hospitalization for brain tumor surgery until 60 postoperative days. Adrenal insufficiency was confirmed based on abnormal serum concentrations of cortisol and inadequate response to the ACTH stimulation test [18].

### 2.3. Surgical Technique

All THAs were performed by three senior surgeons using the modified Watson–Jones anterolateral approach. After resecting the femoral head, medialization was carried out by reaming toward the acetabular fossa until the floor was exposed. Acetabular components were all press-fitted with targets of 40°–45° inclination and 15°–20° anteversion. Dome screws were used only if insufficient press-fitting was perceived during cup insertion. Ceramic-on-ceramic articulation was used in all operations. The femoral procedure was carried out to insert cementless implants of the desired size measured via preoperative templating. The most frequently used femoral prostheses were Bencox (Corentec, Cheon-An, South Korea), S-ROM (DePuy, Warsaw, IN, USA), Trilock (DePuy, Warsaw, IN, USA), and Corail (DePuy).

### 2.4. Postoperative Management

Routine management of venous thromboembolism (VT) prophylaxis was performed after THA. Intermittent pneumatic compression (IPC) devices were applied to all patients after surgery. Aspirin (100 mg) was prescribed from the second postoperative day until 6 weeks, except for patients with bleeding disorders. For those with concurrent use of other antiplatelet drugs or anticoagulants, these drugs were restarted immediately. Patients were encouraged to start walking with a gait support on the first postoperative day. The routine discharge was planned between 4 and 6 days after surgery, depending on the preference of the patient. Discharge was delayed if the postoperative recovery was obviously late or additional medical treatment was necessary.

### 2.5. Clinical Evaluation

The routine clinical visits were scheduled at 2, 6, and 12 months postoperatively and annually thereafter. At each visit, patients were asked about the pain and any uncomfortable feeling on their hips. Physical examinations were performed to check the ROM, and to detect abnormal findings or complications. The occurrence of postoperative dislocation, PJI, PFF, aseptic loosening of implant, and prosthesis failure were considered major surgical complications [19]. The combined ROM was calculated as the sum of degrees in extension to flexion, internal-to-external rotation, and adduction to abduction. We assessed clinical outcomes using the Harris Hip Score (HHS) system and the University of California, Los Angeles (UCLA) activity scale. HHS was subdivided into pain (44 points), functional (47 points), ROM (5 points), and deformity scores (4 points). HHSs of ≥90 points were defined as excellent, those of 80–89 points were defined as good, those of 70–79 points were defined as fair, and those of <70 points were defined as poor [20]. Patients were also asked if they were satisfied with the surgical result for each hip.

### 2.6. Radiographic Evaluation

All radiographic measurements were analyzed twice each by two orthopedic surgeons who had not participated in the index surgery. A standard anteroposterior hip radiograph and a cross-table lateral image obtained on the third postoperative day were considered as the baseline. The images obtained at the last clinical visit were used for the final measurements. Radiolucent lines with a width of >2 mm around the components were considered meaningful. Radiolucency near the acetabular component was determined using the DeLee and Charnley zonal classification [21]. Acetabular loosening was defined as the presence of migration, change of >2° in the angle of the implant, or complete radiolucency around the hemispheric cup [22]. Femoral radiolucency was evaluated using the zonal system of Gruen et al. [23]. Subsidence of the femoral stem of >3 mm was considered meaningful. The modes of stem fixation were classified as bone ingrown, fibrous stable, or loose, according to the classification of Engh et al. [24]. The severity of heterotopic ossification (HO) was determined using the criteria of Brooker et al. [25].

### 2.7. Statistical Analyses

Patients with brain tumors and the control group were matched at a ratio of 1:2 using the propensity score generated by logistic regression. Improvement in the clinical score for each group was determined using paired *t* tests. Continuous variables were compared between groups using Student’s t tests or Wilcoxon rank-sum tests. Chi-squared or Fisher’s exact tests were utilized to compare differences in the distribution of categorical values between groups. Implant survivorships were estimated by Kaplan–Meier survival analyses with the endpoint of revision for any reason, and intergroup differences were determined using log-rank tests. All statistical analyses were performed using SPSS Statistics, version 25.0 (IBM Corp., Armonk, NY). In all analyses, a *P*-value of <0.05 was taken to indicate statistical significance.

## 3. Results

The average time interval from brain surgery to THA was 3.2 (range, 1 to 8) years. The most common brain tumor pathology was meningioma (27%), followed by craniopharyngioma (19%) and pituitary adenoma (12%) (Table 1). There were six malignant tumors (23%), including astrocytoma, glioblastoma multiforme, and germ cell tumors. The most frequent location was the sellar region (46%). All patients received intravenous dexamethasone during the perioperative period of neurosurgery. Prednisolone was prescribed in 15 patients (58%) and hydrocortisone was given in 6 patients (23%). The dose and duration of each corticosteroid therapy are listed in Table 2. After removal of the tumor, additional radiotherapy was performed in nine patients (34%), gamma knife surgery was done in four (15%), and chemotherapy was conducted in three patients (12%).

At the time of THA, 14 patients (54%) had at least one neurological disorder, and 12 (46%) required exogenous corticosteroid replacement for adrenal insufficiency. The mean age at THA was 39.5 (range, 19–67) years and the mean duration of follow-up after THA was 7.4 years (range, 2–15 years) (Table 3). There were no significant differences in demographic characteristics between the two matched groups except for the etiologies of ONFH. The characteristics of surgical procedures were balanced between the two groups (Table 4).

### 3.1. Clinical Outcomes

In both groups, HHS and UCLA activity score significantly improved after THA (*P*-value < 0.001). In the final evaluation, patients with brain tumors had a lower HHS compared to the control group (mean, 80.2 and 89.0, respectively; *P*-value = 0.002) (Table 5). Functional scores were lower in the brain tumor group than those of the control group (mean, 33.2 and 40.5, respectively; *P* = 0.001), whereas pain scores were not significantly different between the brain tumor group and the control group (mean, 38.3 and 40.0, respectively; *P*-value = 0.168). Postoperative UCLA activity scores were lower in the brain tumor group than the control group (median, 5 and 6, respectively; *P*-value < 0.001). Four patients (15.4%) in the brain tumor group experienced postoperative delirium, whereas none had postoperative delirium in the control group (*P*-value = 0.010). Patients with brain tumors had longer hospital stays after THA than the control group (mean, 8.4 and 6.4 days, respectively; *P*-value = 0.005). Patient-reported satisfaction rates (91% and 93%, respectively; *P*-value = 1.000) and postoperative combined ROM (mean, 228.5° and 232.0°, respectively; *P*-value = 0.513) measured at the last clinical visits were similar between the brain tumor group and the control group.

**Figure 2:**
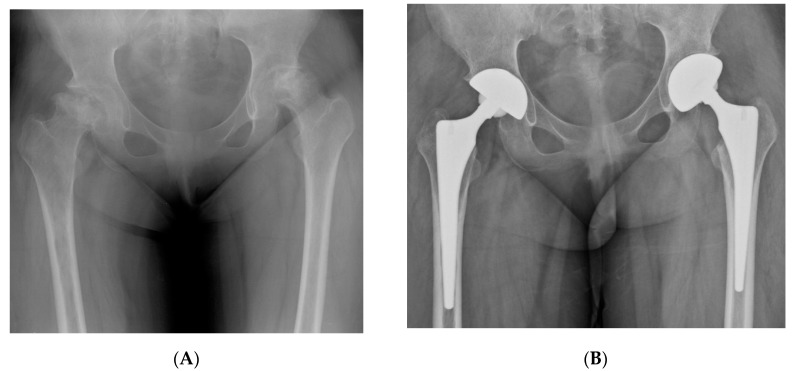

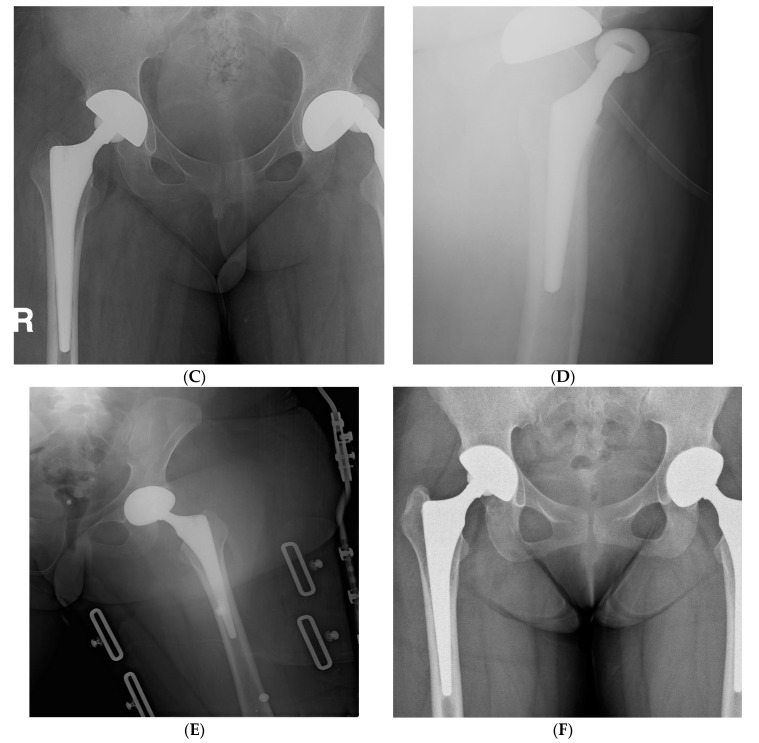
(**A**,**B**) Preoperative and postoperative hip radiographs of a 33-year-old woman with cognitive dysfunction and visual disturbance after removal of recurrent craniopharyngioma. She underwent simultaneous bilateral THA due to ONFH. (**C**,**D**) Radiographs of the same woman on the 15th postoperative day, when she visited the emergency department due to severe pain in the left hip. Anteroposterior and lateral images demonstrated anterior hip dislocation. (**E**) Hip radiograph after performing closed reduction followed by abduction brace application under general anesthesia. (**F**) Eight-year postoperative radiograph showed stable implant fixations.

There were no cases of 90-day mortality in either group, while one unplanned readmission was identified in the brain tumor group; the patient was a 33-year-old woman with moderate cognitive impairment and visual loss in the right eye after resection of craniopharyngioma in the suprasellar region. She developed a non-traumatic hip dislocation on the 15th postoperative day after THA, which was treated successfully by closed reduction and abduction brace application (Figure 2). A PFF (Vancouver B2) in the control group was managed by isolated stem revision using a long modular stem, while no reoperations were performed in the brain tumor group. Symptomatic deep vein thrombosis (DVT) and pulmonary embolism (PE) were not observed in both groups. Overall major complication rates were 2.9% in the brain tumor group and 1.5% in the control group (*P*-value = 1.000).

### 3.2. Radiographic Outcomes

**Figure 3:**
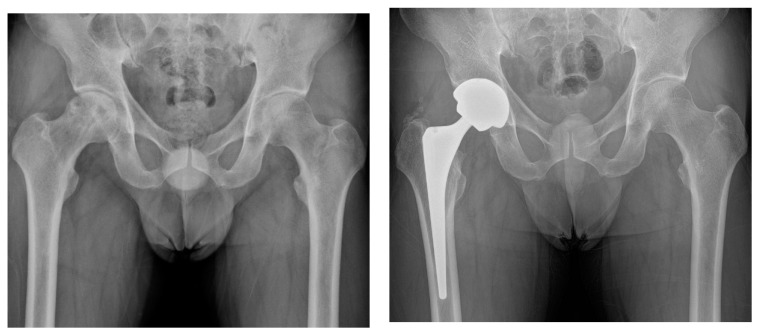
(**A**) Preoperative hip radiograph of a 41-year-old man who had undergone surgical removal of meningioma in the right frontal lobe 4 years prior to THA, demonstrating collapsed osteonecrosis of the right femoral head. (**B**) Five-year postoperative radiograph demonstrating stable implants with Brooker grade 2 heterotopic ossification. The patient had no pain and was satisfied with the outcome.

The rates of the appearance of radiolucent lines in radiographs around the acetabular cup were 5.9% and 4.4% in the brain tumor and control groups, respectively (*P*-value = 1.000); they were 8.8% and 5.9% around the femoral stem (*P*-value = 0.683) (Table 6). There was no complete radiolucency in either group, suggesting acetabular loosening. All femoral radiolucent lines were confined to one or two zones. More HO was observed in patients with brain tumors compared to in the controls (32.4% and 10.3%, respectively; *P*-value = 0.006), all of which were classified as Brooker grade 1 or 2 (Figure 3).

### 3.3. Revision-Free Survivorships

**Figure 4:**
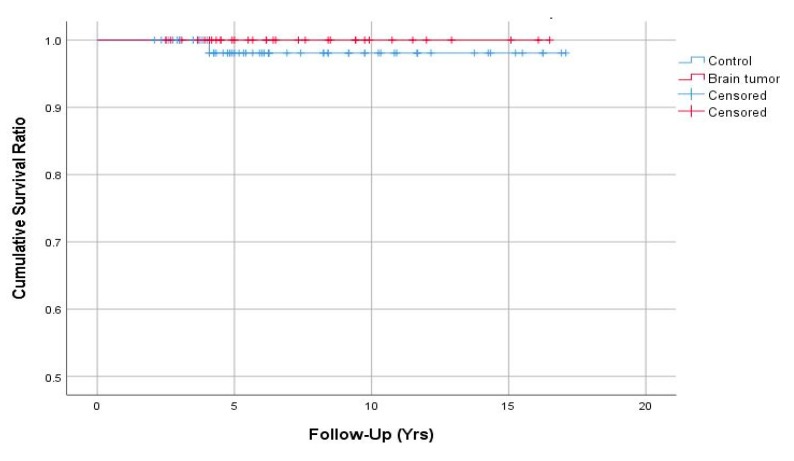
Kaplan–Meier survival curves with the endpoint of revision for any reason.

Implant survivorships with the endpoint of revision for any reason were 100% in the brain tumor group and 98.1% (95% confidence interval, 94.4–100%) in the control group at 7 years (Figure 4). There were no significant differences in the survival estimates at 7 years between the two groups (log-rank, *P*-value = 0.455).

## 4. Discussion

Corticosteroids are the leading cause of non-traumatic ONFH [26]. Despite routine exposure of patients undergoing brain surgery to high-dose corticosteroids, there have been few studies on corticosteroid-induced ONFH in this population [27,28]. To the best of our knowledge, this is the first study to analyze the outcomes of THA in patients with steroid-induced ONFH following surgical treatment of primary brain tumors. Although we assumed higher incidence of complications after THA in the brain tumor group, the major complication rate was not significantly higher compared to in the control group. Hip pain was significantly relieved after THA, and over 90% of brain tumor patients were satisfied with the results. However, it should be noted that more postoperative delirium, longer hospital stay, and lower functional outcomes were identified in the patients with brain tumors.

Several different pathologies of brain tumors were identified in the present study. Although the most common type was meningioma, nearly half of the tumors (pituitary adenoma, craniopharyngioma, germinoma, and dermoid cyst) involved the sella turcica. A higher prevalence of HPA axis dysfunction after resection of sellar masses has been documented [6,7,8]. In this study, 92% of patients with resected sellar lesions had adrenal insufficiency at the time of THA. For these patients, additional corticosteroid supplementation was required in the perioperative period to reduce the risk of adrenal crisis [29]. This usually delayed discharge after THA, because a subsequent tapering process was required to return the steroid to its ordinary dose. There have also been concerns regarding the increased risk for PJI in chronic steroid users. A recent meta-analysis that included 37 studies (2,470,827 patients) reported that a history of steroid use was a risk factor for PJI with an odds ratio of 1.88 [12]. However, no cases of PJI were noted in patients with brain tumors in the present study. In addition to steroid use, other patient-related factors are also associated with the occurrence of PJI. The generally young age and fair general medical status of patients with brain tumors were thought to have had protective effects against the development of PJI.

Seizures and cognitive impairments are the two dominant neurological symptoms in patients with primary brain tumors [30]. Temporal lobe tumors usually impair memory, learning, and language functions, while frontal lobe lesions can cause behavioral and emotional changes [31]. These conditions can affect patient compliance after THA, which can delay functional recovery, and result in longer hospital stays. In the present study, patients with brain tumors had a poorer postoperative HHS. When the HHS was subdivided into pain and functional scales, there were no differences in pain scores between the brain tumor patients and the controls. Lower functional scores in the brain tumor patients were responsible for the lower postoperative HHS. In addition, patients with brain tumors showed less physical activity after THA. In this regard, after performing THA in these patients, it is necessary to focus on efforts to increase functional outcomes through individualized rehabilitation programs.

Postoperative delirium is reported to be more common in neurosurgical patients than in the general population [32]. In this study, the incidence of delirium after THA was 15% in the brain tumor group, which was significantly higher than that in the control group. Postoperative delirium is often extremely difficult to manage and may require admission to a neurointensive care unit in neurosurgical patients. Therefore, prevention and early detection of delirium are crucial in patents with brain tumors. Family members and medical caregivers should communicate clearly and consistently with patients after surgery, provide emotional support, and create an unambiguous hospital environment. Antipsychotic drugs can be also beneficial in reducing the incidence and severity of delirium [33].

One of the common aspects of neurosurgery and orthopaedic surgery is the high incidence of postoperative thromboembolism. Although there was no symptomatic DVT or PE found in this study, patients with glioma are considered to be at higher risk of developing VT after orthopaedic surgery. Therefore, it is mandatory to perform thorough VT prophylaxis including mechanical compression (e.g., elastic stockings and IPC devices), pharmacologic prophylaxis, and early mobilization in patients with brain tumors undergoing THA [34,35].

Nevertheless, neurological morbidities did not give rise to differences in the rates of major complications in THA. A recent multicenter study reported that the odds ratio of dislocation after THA was 3.9 in patients with neurological disorders (cognitive, motor, or psychiatric disability) [14]. Although there are some differences between studies, the incidence of dislocation after contemporary primary THA is approximately 1.5–2% [19,36,37]. In our brain tumor group, the incidence of dislocation was 2.9%, which is not markedly different from that of the general population undergoing THA. This may have been because we used the anterolateral approach in all cases. Several studies have shown that the posterior approach significantly increases the risk of postoperative dislocation [38,39]. Therefore, further studies on larger numbers of cases with different approaches are needed to determine whether the risk is increased in these patients. There may also be concerns regarding the increased risk for PFF in patients with cognitive dysfunction after THA [16]. However, PFF was not observed in patients with brain tumors in the present study. This result can be partially explained by the lower postoperative activity levels in the brain tumor patients than in the controls. Given the incidence of major complications not higher than the general population, THA may be recommended without hesitation in brain tumor patients with severe hip pain for ONFH.

In final radiographs, there were no complete radiolucent lines or position changes, suggesting aseptic loosening of implant in either group. The frequencies of the appearance of partial radiolucent lines around the acetabular and femoral components were balanced between the two groups. On the other hand, the incidence of HO was higher in the brain tumor group. Although several intrinsic substances (e.g., growth factors, neuropeptides, and hormones) promote bone formation after traumatic brain injury, the association between brain tumors and HO is not well understood [40]. Prolonged immobility is another major factor involved in the development of HO [41]. Although we recommended all patients to mobilize and commence self-ambulation on the first postoperative day after THA, poor compliance and decreased activity levels in patients with brain tumors probably contributed to the development of HO. Fortunately, all HOs in the brain tumor group were classified as grade 1 or 2, and the postoperative pain and ROM seemed not to be affected by these conditions.

The present study had several limitations. First, the number of patients in the brain tumor group was too small to compare the frequency of each postoperative complication with that in the control group. However, considering the low prevalence of primary brain tumors and subsequent development of ONFH, collecting data on larger numbers of cases would not be feasible at a single institution. A multicenter study is needed in the future to overcome this limitation. Second, the study population included only patients undergoing THA at the same institution where neurosurgery was performed. Therefore, the exact incidence of ONFH after brain tumor surgery could not be confirmed in this study. Considering the number of ONFHs managed non-operatively or treated in other institutions, the actual incidence would be higher than 0.3%. However, using this methodology, it was relatively accurate in determining the diagnosis, corticosteroid dose, and neurological status after surgical treatment of brain tumors. Third, the exact causal relationship and risk factors for developing ONFH in patients with brain tumors are unclear due to the small sample size and retrospective study design. They should be identified using a larger cohort with prospective study design in the future research. Finally, the mean follow-up duration of 7.4 years was relatively short, particularly for young patients with brain tumors.

## 5. Conclusions

THA performed in steroid-induced ONFH after surgical removal of primary brain tumors demonstrated favorable clinical results with high patient satisfaction. Postoperative pain scores, major complication rates, and implant survivorships were similar between the brain tumor and control groups at 7 years. Our findings suggest that THA can be safely performed to reduce hip pain in patients with corticosteroid-induced ONFH after surgical treatment of primary brain tumors.

## Figures and Tables

**Table 1:** Characteristics of brain tumor and neurological morbidities after surgical removal.

Characteristics	Total (*n* = 26)
Pathology	
Meningioma	7 (27%)
Craniopharyngioma	5 (19%)
Pituitary adenoma	3 (12%)
Germinoma	2 (7.7%)
Others *	9 (35%)
Location	
Sella turcica	12 (46%)
Frontal lobe	6 (23%)
Temporal lobe	3 (12%)
Others †	5 (19%)
Neurological sequelae	
Seizure	8 (31%)
Cognitive impairment	5 (19%)
Visual disturbance	5 (19%)
Hemiparesis	2 (7.7%)
Hearing loss	1 (3.8%)

Values are given as the number of patients with the percentage in parentheses. * Others included astrocytoma, glioblastoma multiforme, cavernous angioma, acoustic schwannoma, dermoid cyst, hemangioblastoma, and pineal parenchymal tumors. † Others included parietal lobe, cerebellum, and skull base other than sella turcica.

**Table 2:** Daily dose and duration of corticosteroid therapy in the perioperative period of brain tumor surgery.

Corticosteroids	Total (*n* = 26)
Dexamethasone	
Daily dose (mg)	16.0 ± 8.2
Duration (days)	14.3 ± 9.1
Prednisolone	
Daily dose (mg)	17.6 ± 8.8
Duration (days)	11.7 ± 6.1
Hydrocortisone	
Daily dose (mg)	76.9 ± 19.8
Duration (days)	3.9 ± 1.6

Values are given as the mean ± standard deviation.

**Table 3:** Demographic data of the two matched groups.

Demographics	Brain Tumor	Control	*P*-Value
Number of patients (hips)	26 (34)	52 (68)	
Age * (years)	39.5 ± 11.3	40.7 ± 11.9	0.414
Female patients †	15 (58%)	30 (58%)	1.000
Body mass index * (kg/m^2^)	25.3 ± 5.6	24.2 ± 3.4	0.306
American Society of Anesthesiologists score †			1.000
1	5 (19%)	11 (21%)	
2	20 (77%)	39 (75%)	
3	1 (3.8%)	2 (3.8%)	
Etiology of ONFH ‡			<0.001
Corticosteroid	34 (100%)	21 (31%)	
Trauma		23 (34%)	
Alcohol		9 (13%)	
Others or unknown		15 (22%)	
Preoperative Harris Hip Score *	42.8 ± 16.7	44.7 ± 15.8	0.567
Preoperative UCLA activity score §	3 (1–5)	3 (1–6)	0.721
Preoperative combined ROM * (°)	150.2 ± 39.4	153.9 ± 36.1	0.633
Duration of follow-up * (years)	7.4 ± 3.9	7.3 ± 4.2	0.870

* Values are given as the mean ± standard deviation. † Values are given as the number of patients with the percentage in parentheses. ‡ Values are given as the number of hips with the percentage in parentheses. § Values are given as the median with the range in parentheses. UCLA = University of California, Los Angeles. ROM = range of motion.

**Table 4:** Surgical characteristics of THA.

Characteristics	Brain Tumor (*n* = 34)	Control (*n* = 68)	*P*-Value
Spinal anesthesia *	28 (82%)	62 (91%)	0.208
Cup diameter (mm)	51.0 ± 2.9	51.4 ± 3.6	0.560
Head diameter *			0.548
28 mm	3 (8.8%)	10 (15%)	
32 mm	11 (32%)	25 (37%)	
36 mm	20 (59%)	33 (49%)	
Femoral stem *			0.733
Bencox	22 (65%)	35 (52%)	
S-ROM	5 (15%)	11 (16%)	
Trilock	2 (5.9%)	8 (12%)	
Corail	2 (5.9%)	8 (12%)	
Others	3 (8.8%)	6 (8.8%)	
Operation time (min)	81.2 ± 11.6	79.8 ± 14.5	0.776

* Values are given as the number of hips with the percentage in parentheses. Other values are given as the mean ± standard deviation.

**Table 5:** Clinical outcomes of THA.

Outcomes	Brain Tumor (*n* = 34)	Control (*n* = 68)	*P*-Value
Postoperative Harris Hip Score *	80.2 ± 13.8	89.0 ± 9.9	0.002
Pain score	38.3 ± 6.1	40.0 ± 5.3	0.168
Functional score	33.2 ± 11.0	40.5 ± 7.3	0.001
Ratings of Harris Hip Score			0.007
Excellent	10 (29%)	38 (56%)	
Good	8 (24%)	18 (27%)	
Fair	7 (21%)	8 (12%)	
Poor	9 (27%)	4 (5.9%)	
Postoperative UCLA activity score †	5 (3–7)	6 (4–9)	<0.001
Number of satisfactory hips	31 (91%)	63 (93%)	1.000
Postoperative combined ROM * (°)	228.5 ± 25.8	232.0 ± 24.6	0.513
Length of hospital stay * (days)	8.4 ± 3.7	6.4 ± 1.6	0.005
Major surgical complications	1 (2.9%)	1 (2.9%)	1.000
Periprosthetic femoral fracture	0 (0%)	1 (2.9%)	1.000
Dislocation	1 (2.9%)	0 (0%)	0.333
Periprosthetic joint infection	0 (0%)	0 (0%)	1.000
Aseptic loosening	0 (0%)	0 (0%)	1.000
Reoperation	0 (0%)	1 (1.5%)	1.000

* Values are given as the mean ± standard deviation. † Values are given as the median with the range in parentheses. Other values are given as the number of hips with the percentage in parentheses. UCLA = University of California, Los Angeles. ROM = range of motion.

**Table 6:** Radiographic outcomes of THA.

Outcomes	Brain Tumor (*n* = 34)	Control (*n* = 68)	*P*-Value
Radiolucency around the cup	2 (5.9%)	3 (4.4%)	1.000
Aseptic loosening of the cup	0 (0%)	0 (0%)	1.000
Radiolucency around stem	3 (8.8%)	4 (5.9%)	0.683
Stem subsidence	2 (5.9%)	1 (1.5%)	0.257
Stem stability			1.000
Bone ingrowth	33 (97%)	67 (99%)	
Fibrous stable	1 (2.9%)	1 (1.5%)	
Loosening	0 (0%)	0 (0%)	
Heterotopic ossification	11 (32%)	7 (10%)	0.006

Values are given as the number of hips with the percentage in parentheses.

## References

[1] Dietrich J., Rao K., Pastorino S., Kesari S. (2011). Corticosteroids in brain cancer patients: Benefits and pitfalls. Expert Rev. Clin. Pharmacol..

[2] Murayi R., Chittiboina P. (2016). Glucocorticoids in the management of peritumoral brain edema: A review of molecular mechanisms. Child’s Nerv. Syst..

[3] McClelland S., Long D.M. (2008). Genesis of the use of corticosteroids in the treatment and prevention of brain edema. Neurosurgery.

[4] Roth P., Happold C., Weller M. (2015). Corticosteroid use in neuro-oncology: An update. Neuro-Oncol. Pract..

[5] Kostaras X., Cusano F., Kline G.A., Roa W., Easaw J. (2014). Use of dexamethasone in patients with high-grade glioma: A clinical practice guideline. Currentoncology.

[6] Zada G., Tirosh A., Huang A.P., Laws E.R., Woodmansee W.W. (2013). The postoperative cortisol stress response following transsphenoidal pituitary surgery: A potential screening method for assessing preserved pituitary function. Pituitary.

[7] Jahangiri A., Wagner J.R., Han S.W., Tran M.T., Miller L.M., Chen R., Tom M.W., Ostling L.R., Kunwar S., Blevins L. (2016). Improved versus worsened endocrine function after transsphenoidal surgery for nonfunctional pituitary adenomas: Rate, time course, and radiological analysis. J. Neurosurg..

[8] Agam M.S., Zada G. (2018). Complications associated with transsphenoidal pituitary surgery: Review of the Literature. Neurosurgery.

[9] Lim S.J., Kim S.M., Kim D.W., Moon Y.W., Park Y.S. (2016). Cementless total hip arthroplasty using Biolox(R) delta ceramic-on-ceramic bearing in patients with osteonecrosis of the femoral head. Hip Int..

[10] Kim S.M., Lim S.J., Moon Y.W., Kim Y.T., Ko K.R., Park Y.S. (2013). Cementless modular total hip arthroplasty in patients younger than fifty with femoral head osteonecrosis: Minimum fifteen-year follow-up. J. Arthroplast..

[11] Amiche M.A., Albaum J.M., Tadrous M., Pechlivanoglou P., Levesque L.E., Adachi J.D., Cadarette S.M. (2016). Fracture risk in oral glucocorticoid users: A Bayesian meta-regression leveraging control arms of osteoporosis clinical trials. Osteoporos. Int..

[12] Resende V.A.C., Neto A.C., Nunes C., Andrade R., Espregueira-Mendes J., Lopes S. (2018). Higher age, female gender, osteoarthritis and blood transfusion protect against periprosthetic joint infection in total hip or knee arthroplasties: A systematic review and meta-analysis. Knee Surg. Sports Traumatol. Arthrosc..

[13] Kunutsor S.K., Whitehouse M.R., Blom A.W., Beswick A.D. (2016). Patient-related risk factors for periprosthetic joint infection after total joint arthroplasty: A systematic review and meta-analysis. PLoS ONE.

[14] Fessy M.H., Putman S., Viste A., Isida R., Ramdane N., Ferreira A., Leglise A., Rubens-Duval B., Bonin N., Bonnomet F. (2017). What are the risk factors for dislocation in primary total hip arthroplasty? A multicenter case-control study of 128 unstable and 438 stable hips. Orthop. Traumatol. Surg. Res..

[15] Dawson-Amoah K., Raszewski J., Duplantier N., Waddell B.S. (2018). Dislocation of the hip: A review of types, causes, and treatment. Ochsner J..

[16] Broden C., Mukka S., Muren O., Eisler T., Boden H., Stark A., Skoldenberg O. (2015). High risk of early periprosthetic fractures after primary hip arthroplasty in elderly patients using a cemented, tapered, polished stem. Acta Orthop..

[17] Ganguli M., Dodge H.H., Shen C., DeKosky S.T. (2004). Mild cognitive impairment, amnestic type: An epidemiologic study. Neurology.

[18] Streeten D.H., Anderson G.H., Bonaventura M.M. (1996). The potential for serious consequences from misinterpreting normal responses to the rapid adrenocorticotropin test. J. Clin. Endocrinol. Metab..

[19] Dargel J., Oppermann J., Bruggemann G.P., Eysel P. (2014). Dislocation following total hip replacement. J. Bone Jt. Surg..

[20] Zeng W.N., Liu J.L., Jia X.L., Zhou Q., Yang L., Zhang Y. (2019). Midterm results of total hip arthroplasty in patients with high hip dislocation after suppurative hip arthritis. J. Arthroplast..

[21] DeLee J.G., Charnley J. (1976). Radiological demarcation of cemented sockets in total hip replacement. Clin. Orthop. Relat. Res..

[22] Fredin H., Sanzen L., Sigurdsson B., Unander-Scharin L. (1991). Total hip arthroplasty in high congenital dislocation. 21 hips with a minimum five-year follow-up. J. Bone Jt. Surg..

[23] Gruen T.A., McNeice G.M., Amstutz H.C. (1979). Modes of failure" of cemented stem-type femoral components: A radiographic analysis of loosening. Clin. Orthop. Relat. Res..

[24] Engh C.A., Bobyn J.D., Glassman A.H. (1987). Porous-coated hip replacement. The factors governing bone ingrowth, stress shielding, and clinical results. J. Bone Jt. Surg..

[25] Brooker A.F., Bowerman J.W., Robinson R.A., Riley L.H. (1973). Ectopic ossification following total hip replacement: Incidence and a method of classification. JBJS.

[26] Yoon B.H., Jones L.C., Chen C.H., Cheng E.Y., Cui Q., Drescher W., Fukushima W., Gangji V., Goodman S.B., Ha Y.C. (2019). Etiologic Classification Criteria of ARCO on Femoral Head Osteonecrosis Part 1: Glucocorticoid-Associated Osteonecrosis. J. Arthroplast..

[27] Wong G.K., Poon W.S., Chiu K.H. (2005). Steroid-induced avascular necrosis of the hip in neurosurgical patients: Epidemiological study. ANZ J. Surg..

[28] Uppal J., Burbridge B., Arnason T. (2019). Bilateral osteonecrosis of the hip in panhypopituitarism. BMJ Case Rep. CP.

[29] Liu M.M., Reidy A.B., Saatee S., Collard C.D. (2017). Perioperative Steroid Management Approaches Based on Current Evidence. Anesthesiology.

[30] Posti J.P., Bori M., Kauko T., Sankinen M., Nordberg J., Rahi M., Frantzen J., Vuorinen V., Sipila J.O. (2015). Presenting symptoms of glioma in adults. Acta Neurol. Scand..

[31] Ali F.S., Hussain M.R., Gutierrez C., Demireva P., Ballester L.Y., Zhu J.J., Blanco A., Esquenazi Y. (2018). Cognitive disability in adult patients with brain tumors. Cancer Treat. Rev..

[32] Flanigan P.M., Jahangiri A., Weinstein D., Dayani F., Chandra A., Kanungo I., Choi S., Sankaran S., Molinaro A.M., McDermott M.W. (2018). Postoperative delirium in glioblastoma patients: Risk factors and prognostic implications. Neurosurgery.

[33] Ganau M., Lavinio A., Prisco L. (2018). Delirium and agitation in traumatic brain injury patients: An update on pathological hypotheses and treatment options. Minerva Anestesiol..

[34] Ganau M., Prisco L., Cebula H., Todeschi J., Abid H., Ligarotti G., Pop R., Proust F., Chibbaro S. (2017). Risk of deep vein thrombosis in neurosurgery: State of the art on prophylaxis protocols and best clinical practices. J. Clin. Neurosci..

[35] Chibbaro S., Cebula H., Todeschi J., Fricia M., Vigouroux D., Abid H., Kourbanhoussen H., Pop R., Nannavecchia B., Gubian A. (2018). Evolution of prophylaxis protocols for venous thromboembolism in neurosurgery: Results from a prospective comparative study on low-molecular-weight heparin, elastic stockings, and intermittent pneumatic compression devices. World Neurosurg..

[36] Buckland A.J., Puvanesarajah V., Vigdorchik J., Schwarzkopf R., Jain A., Klineberg E.O., Hart R.A., Callaghan J.J., Hassanzadeh H. (2017). Dislocation of a primary total hip arthroplasty is more common in patients with a lumbar spinal fusion. Bone Jt. J..

[37] Jameson S.S., Lees D., James P., Serrano-Pedraza I., Partington P.F., Muller S.D., Meek R.M., Reed M.R. (2011). Lower rates of dislocation with increased femoral head size after primary total hip replacement: A five-year analysis of NHS patients in England. J. Bone Jt. Surg..

[38] Zijlstra W.P., DeHartog B., VanSteenbergen L.N., Scheurs B.W., Nelissen R. (2017). Effect of femoral head size and surgical approach on risk of revision for dislocation after total hip arthroplasty: An analysis of 166,231 procedures in the Dutch Arthroplasty Register (LROI). Acta Orthop..

[39] Sheth D., Cafri G., Inacio M.C., Paxton E.W., Namba R.S. (2015). Anterior and anterolateral approaches for THA are associated with lower dislocation risk without higher revision risk. Clin. Orthop. Relat. Res..

[40] Huang H., Cheng W.X., Hu Y.P., Chen J.H., Zheng Z.T., Zhang P. (2018). Relationship between heterotopic ossification and traumatic brain injury: Why severe traumatic brain injury increases the risk of heterotopic ossification. J. Orthop. Transl..

[41] Hudson S.J., Brett S.J. (2006). Heterotopic ossification–a long-term consequence of prolonged immobility. Crit. Care.

